# Amino-acid PET as a prognostic tool after post Stupp protocol temozolomide therapy in high-grade glioma patients

**DOI:** 10.1007/s11060-024-04722-2

**Published:** 2024-06-06

**Authors:** Adeline Zinsz, Shamimeh Ahrari, Jason Becker, Ali Mortada, Veronique Roch, Louis Doriat, Matthieu Santi, Marie Blonski, Luc Taillandier, Timothée Zaragori, Antoine Verger

**Affiliations:** 1 Department of Nuclear Medicine and Nancyclotep Imaging Platform, Université de Lorraine, CHRU Nancy, 54000 Nancy, France; 2 Université de Lorraine, IADI, INSERM U1254, F-54000 Nancy, France; 3 Department of Neuro-Oncology, Université de Lorraine, CHRU Nancy, 54000 Nancy, France; 4 Nuclear Medicine Department, CHRU Nancy, Rue du Morvan, 54500 Vandoeuvre Les Nancy, France

**Keywords:** Glioma, PET, Temozolomide, Survivals, Stupp, Treatment monitoring

## Abstract

**Purpose:**

This study aimed to evaluate the prognostic performance of amino-acid PET in high-grade gliomas (HGG) patients at the time of temozolomide (TMZ) treatment discontinuation, after the Stupp protocol.

**Methods:**

The analysis included consecutive HGG patients with dynamic [18F]FDOPA PET imaging within 3 months of the end of TMZ therapy, post-Stupp protocol. Static and dynamic PET parameters, responses to RANO criteria for MRI and clinical and histo-molecular factors were correlated to progression-free (PFS).

**Results:**

Thirty-two patients (59.4 [54.0;67.6] years old, 13 (41%) women) were included. Static PET parameters peak tumor-to-background ratio and metabolic tumor volume (respective thresholds of 1.9 and 1.5 mL) showed the best 84% accuracies for predicting PFS at 6 months (p = 0.02). These static PET parameters were also independent predictor of PFS in multivariate analysis (p ≤ 0.05).

**Conclusion:**

In HGG patients having undergone a Stupp protocol, the absence of significant PET uptake after TMZ constitutes a favorable prognostic factor.

**Supplementary Information:**

The online version contains supplementary material available at 10.1007/s11060-024-04722-2.

## Introduction

The treatment of high-grade glioma (HGG) is based on total surgical resection when possible [1] followed by the Stupp protocol, which combines radiotherapy with concomitant and subsequently adjuvant chemotherapy with Temozolomide (TMZ) [2]. Although the treatment strategy is adapted to each individual, patients in good general and neurological condition are offered adjuvant TMZ maintenance cycles following the traditional Stupp protocol [3]. Nevertheless, 60–75% of glioblastoma patients derive no benefit from TMZ [4], and 15–20% develop clinically toxicity [4]. Diagnostic tools for monitoring TMZ treatment are therefore required.

Amino-acid positron emission tomography (PET) is a recommended adjunct to magnetic resonance imaging (MRI), all along the clinical course of glioma [5]. Whilst the current literature on the value of amino acid PET at initial diagnosis, treatment planning and detection of recurrences is expanding rapidly [6], studies investigating its impact on treatment follow-up remain scarce. Importantly, few studies have to date reported results of amino-acid PET planning at the time of TMZ discontinuation in patients who had undergone a Stupp protocol and none examined the amino-acid PET radiotracer, 3,4-dihydroxy-6-[18F]-fluoro-L-phenylalanine ([18F]FDOPA) [7].

This study therefore aimed to evaluate the prognostic performances of [18F]FDOPA PET at the time of TMZ discontinuation in HGG patients that completed the Stupp protocol.

## Material and methods

### Population

We retrospectively included consecutive HGG patients referred for dynamic [18F]FDOPA PET between December 2018 to February 2022 within 3 months after TMZ discontinuation following a Stupp protocol. For all these patients, the PET was prescribed to evaluate the clinical response to TMZ. All glioma lesions were reclassified by histo-molecular diagnostics according to the WHO 2021 brain tumor classification [8]. The decisions to treat with the Stupp protocol and to discontinue TMZ were taken at multidisciplinary neuro-oncological board meetings. Progression free survival (PFS) was defined as the time intervals from the end of TMZ treatment to progression, according to the RANO criteria [9].

### [18F]FDOPA PET imaging

Patients fasted for at least 4 h before the scan on a digital PET/CT camera (Vereos; Philips®). From December 2018 to September 2020, patients received Carbidopa 1 h before the radiotracer injection to increase striatal dopaminergic activity and tumor uptake. A 30 min dynamic PET acquisition was performed following the injection of 2 MBq ^18^F-FDOPA/kg of body weight. One static image based on the last 20 min of acquisitions and 30 frames of 1 min each for dynamic images were reconstructed using an OSEM 3D algorithm (2 iterations, 10 subsets, 256 × 256 × 164 voxels of 1 × 1 × 1 mm^3^) and no post-filtering. Details of reconstruction processes are available elsewhere [10].

### Semi-quantitative analysis of imaging

All dynamic [18F]FDOPA PET images were co-registered with static PET images using a rigid transform.

On static PET images, a bounding box was first delineated for each lesion using the LifeX software [11] to specifically avoid including regions with physiological uptake such as the striatum. PET segmentation was performed semi-automatically using a 1.6-fold SUV_mean_ healthy brain threshold [12–14]. Tumor-to-background (TBR) and tumor-to-striatum (TSR) ratios with respectively SUV_max_, SUV_mean_ and SUV_peak_ of the tumor volume were determined. Time-to-peak (TTP) and slope of uptake value between the 10th and the 30th minute post injection were calculated [12]. To correct for the impact of Carbidopa, static TBR parametric images and normalized region-based time activity curves were generated [15].

All MRI images, at the beginning and at the end of the TMZ therapy. including at least T1-weighted with injection and T2-FLAIR weighted sequences, were reviewed and MRI responses classified as complete or partial, and stable or progressive disease according to the RANO criteria according to reports of experienced neuro-radiologists [9].

All PET final segmentations and MRI classifications were reviewed by an experienced physician (A.V.) to ascertain the quality of the methods applied.

### Statistical analysis

Categorical variables are expressed as percentages and continuous variables as medians [first quartiles; third quartiles]. Receiver operating characteristic curves of static and dynamic PET parameters were calculated for PFS at 6 months with decision cut-offs defined as the point on the curve closest to (0,1). Univariate and multivariate Cox regression analyses were performed to test for associations between PFS and amino-acid PET uptake as well as MRI response assessment and clinical factors, including age, sex, the WHO global status, whether the patient had a biopsy or a subtotal or complete surgery, the WHO 2021 glioma grade, the IDH and the MGMT statutes. A p-value < 0.05 was considered significant. Statistical analyses were performed using R version 4.1.1 (R Foundation for Statistical Computing, Vienna, Austria).

## Results

Thirty-two patients (59.4 [54.0;67.6] years old, 13 (41%) women) having received a median of 6 [6;12] TMZ cycles were finally included. The histo-molecular diagnoses were glioblastoma IDH-wildtype (n = 27, 84.4%), astrocytoma IDH-mutant (n = 4, 12.5%) and diffuse midline glioma, H3k27M-mutant (n = 1, 3.1%). In the 29 patients with MGMT status available, 13 (45%) presented a methylation. PET scans were performed at 21 [11;37] days after the end of TMZ. During follow-up of 32.4 [22.8;37.5] months, 20 patients progressed (7 progressions at 6 months) and 11 patients died. 21 patients (66%) had a significant amino-acid PET uptake (i.e., a metabolic tumor volume, MTV) at the end of TMZ treatment. The median time interval between MRI and amino acid PET was 3 [-13.5;13.25] days. 14 patients (44%) had a complete or partial response by MRI. All detailed characteristics of patients are available in the Supplemental Table 1.

**Table 1:** Diagnostic performances of PET parameters in terms of PFS at 6 months. Bold parameters are significant

	Threshold	AUC	Sensitivity	Specificity	Accuracy	*P*-value
**TBR**_**mean**_	1.78	0.74	0.71	0.80	0.78	0.05
**TBR**_**max**_	2.05	0.78	0.86	0.72	0.75	0.02
**TBR**_**peak**_	1.85	0.79	0.71	0.88	0.84	0.02
TSR_mean_	0.90	0.69	0.88	0.60	0.66	0.15
**TSR**_**max**_	1.06	0.74	0.86	0.60	0.66	0.05
**TSR**_**peak**_	0.92	0.78	0.71	0.76	0.75	0.02
**MTV (mL)**	1.51	0.75	0.71	0.88	0.84	0.04
TTP (min)	26.39	0.55	0.57	0.52	0.53	0.70
Slope (h^−1^)	0.18	0.54	0.57	0.64	0.63	0.79

*AUC: Area under the curve, MTV: metabolic tumor volume, TBR: tumor-to-background ratio; TSR: tumor-to-striatum ratio*

**Fig. 1:**
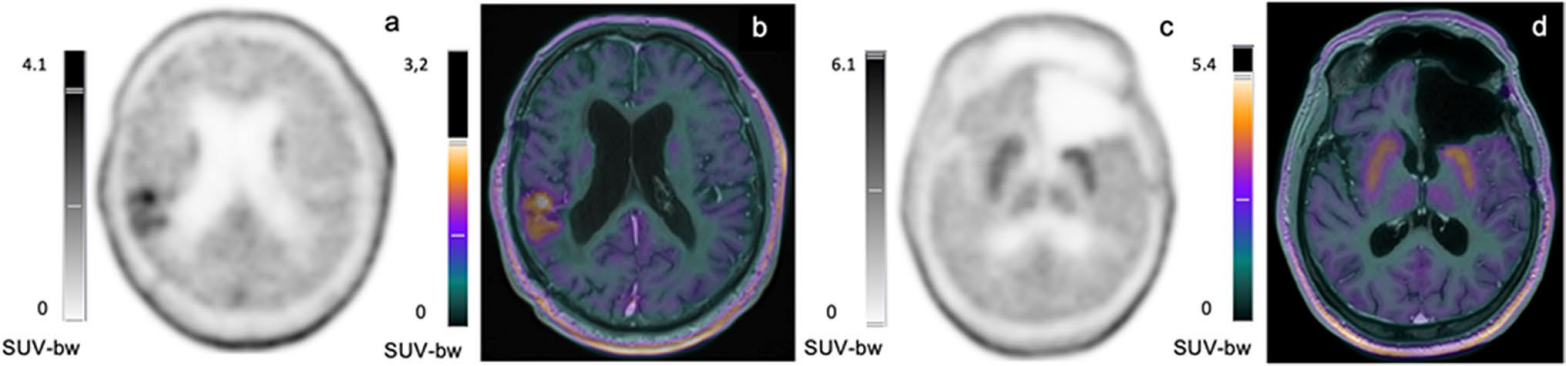
(**a**)(**c**) Static [18F]FDOPA PET images; (**b**)(**d**) Co-registered PET and MRI images (fused). The two left panels are those of an 81-years-old man with an IDH-wildtype glioblastoma with respective PET parameters obtained after 22 TMZ cycles: TBR_peak_: 2.3, MTV: 7.3 mL. Follow-up revealed progression at 3.9 months. The two right panels are those of a 54-years-old man with an IDH-wildtype glioblastoma with respective PET parameters obtained after 6 TMZ cycles: TBR_peak_: 1.5, MTV: 0 ml. Follow-up revealed progression at 12.5 months

All static PET parameters, except TSR_mean_, were significant for the prediction of PFS at 6 months, with TBR_peak_ (1.9 threshold) and MTV (1.5 mL threshold) showing the best accuracies (84%, p = 0.02, Table 1). Among all clinical, MRI and PET parameters, only TBR_peak_ and MTV were significant in univariate Cox model analysis (p ≤ 0.05, Table 2). In multivariate analysis, TBR_peak_ represented the only significant predictor of PFS in the best model (HR = 3.6 [1.1–12.1], p = 0.04) and MTV in the second-best model (HR = 1.3 [1.0–1.6], p = 0.05). Figure 1 shows representative images with and without significant PET uptake. Supplemental Fig. 1 shows Kaplan Meier curves for patients with or without significant amino-acid PET uptake (i.e. with an MTV) at the end of TMZ.

**Table 2:** Univariate Cox model analysis for PFS. Bold parameters are significant

Parameters	*P*-value
**Clinical parameters**	
Age	0.47
Sex	0.58
WHO global status	0.87
Biopsy/Subtotal surgery/ Complete surgery	0.44
WHO 2021 classification	0.22
IDH mutation	0.36
MGMT status	0.49
RANO criteria	0.48
**PET parameters**	
TBR_mean_	0.18
TBR_max_	0.055
** TBR**_**peak**_	0.04
TSR_mean_	0.30
TSR_max_	0.14
TSR_peak_	0.10
** MTV**	0.047
Time-to-peak	0.30
Slope	0.47

*MTV: metabolic tumor volume, PET: Positron emission tomography; RANO: Response Assessment in Neuro-Oncology; TBR: tumor-to-background ratio; TSR: tumor-to-striatum ratio;* *WHO: World health organization*

## Discussion

The present study shows that the absence of significant uptake in [18F]FDOPA PET after cycles of adjuvant TMZ constitutes a favorable prognostic factor.

Several studies have investigated the value of amino-acid PET for treatment follow-up of gliomas [7], but none thus far with the amino-acid [18F]FDOPA radiotracer at the time of TMZ discontinuation, post Stupp protocol. The most complete study to date was performed with ^18^F-fluoro-ethyl-tyrosine ([18F]-FET) in a prospective cohort of 79 glioblastoma patients [16]. In this study, the MTV was a prognostic factor of survival but only prior to any treatment. Another study of 44 glioblastoma patients using ^11^C-methionine ([11C]-MET) showed that a positive MTV, defined as tumor uptake at least two-fold above healthy brain uptake, was an independent prognostic factor of PFS, at the time of adjuvant TMZ discontinuation [17]. This is in line with our current results where the presence of an MTV (of at least 1.5 mL), as well as significant TBR_peak_ PET parameter (threshold at 1.9), were predictors of PFS at 6 months at the time of TMZ discontinuation (Table 1). Moreover, TBR_peak_ or MTV were independent prognostic factors of PFS whilst MRI RANO criteria, clinical and histo-molecular factors were not (Table 2). It is noteworthy that application of the proposed cut-offs (near 2 for TBR_peak_ and 0 mL for MTV) are entirely feasible in routine practice. A positive MTV 1.6-fold greater than the SUV_mean_ healthy brain threshold is reported in the European glioma guidelines for amino-acid PET [13] and was subsequently applied to [18F]FDOPA PET imaging [12] is associated with shorter survival albeit non-significantly mainly due to the low number of patients included (Supplemental Fig. 1). The current study also confirms that dynamic PET parameters provide less prognostic information than static PET parameters in previously treated glioma patients [18].

Our patient population is highly selected because it excludes most HGG patients with recurrences occurring during TMZ adjuvant therapy as part of a Stupp protocol. However, determining the prognosis of patients at the time of TMZ discontinuation is generally a matter for debate at multidisciplinary neuro-oncology board meetings, reinforcing the critical importance of a prognostic biomarker in these types of patients.

To conclude, performing amino-acid PET in HGG patients after TMZ therapy is a useful prognostic tool which should help clinicians decide whether or not to discontinue treatment based on the significant uptake observed on PET.

### Supplementary Information

Below is the link to the electronic supplementary material.

Supplementary file1 (TIF 806 KB)

Supplementary file2 (XLSX 20 KB)

## Data Availability

The datasets generated during and/or analyzed during the current study are available in the Supplemental Table 1.
