# Supplementary figures and images for: Amino-acid PET as a prognostic tool after post Stupp protocol temozolomide therapy in high-grade glioma patients
Source: J Neurooncol. 2024 Jun 6;169(2):241–5. doi: 10.1007/s11060-024-04722-2 (PMC11341581; doi:10.1007/s11060-024-04722-2)

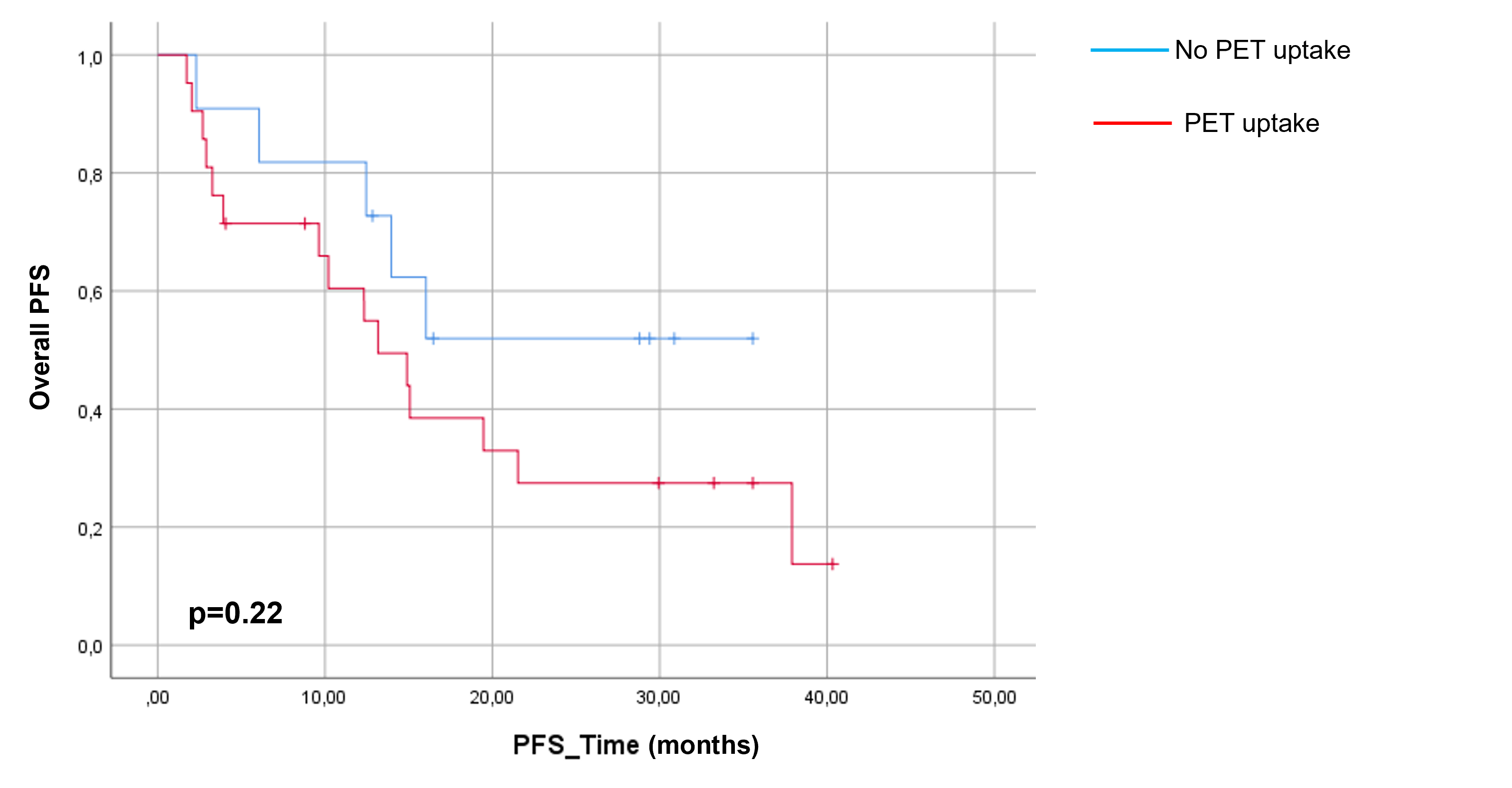

Supplement: Supplementary file 1 — Supplementary file1 (TIF 806 KB) [file 11060_2024_4722_MOESM1_ESM.tif]
